# Comparison of the introduction of consistencies in complementary feeding introduction between preterm and full-term newborns - Cohort from 0 to 12 months

**DOI:** 10.1590/2317-1782/20232022315en

**Published:** 2023-10-13

**Authors:** Hellen Nataly Correia Lagos Guimarães, Andriéllen Marciniak, Lívia dos Santos Paula, Sheila Tamanini de Almeida, Adriane Celli

**Affiliations:** 1 Programa de Pós-graduação em Saúde da Criança e do Adolescente, Universidade Federal do Paraná - UFPR - Curitiba (PR), Brasil.; 2 Departamento de Fonoaudiologia, Universidade Federal de Ciências da Saúde de Porto Alegre - UFCSPA - Porto Alegre (RS), Brasil.

**Keywords:** Feeding Behavior, Complementary Food, Breastfeeding, Growth, Premature Newborn

## Abstract

**Purpose:**

To compare the introduction of consistencies during the period of complementary feeding of preterm and full-term newborns up to 12 months of life, as well as to evaluate the presence of oral motor dysfunction and its relation to difficulty in introducing food consistencies in these groups.

**Methods:**

This is an observational, analytical, cohort study, with ambispective data collection, carried out at the Municipal Department of Health of Mafra, state of Santa Catarina, Brazil. The study sample consisted of 87 newborns, 41 full-term and 46 preterm. While data was collected, interviews were held with the mothers/guardians. The anthropometric assessment was carried out by a nutritionist by measuring body weight, length, and head circumference, followed by assessment of oral and functional motor skills by the adapted Clinical Evaluation Protocol of Pediatric Dysphagia (PAD-PED), assessment of breastfeeding and neuropsychomotor development, and assessment of the presence of maternal depression and psychological risk of children with up to 12 months of corrected age.

**Results:**

We verified oral motor dysfunction in 15 newborns, in both groups, in the liquid consistency in the first assessment, persisting in two cases in the full-term newborns and in three cases in the preterm infants, in the last assessment for the solid consistency.

**Conclusion:**

We observed no difference in the introduction of food consistencies between groups. Breastfeeding was more frequent in newborns in the first assessment and similar in other assessments. Regarding the predictors for oral motor dysfunction, bottle feeding increased the odds by about seven times and invasive oral procedures by about six times.

## INTRODUCTION

Competence for oral feeding of preterm infants is considered an essential requirement for hospital discharge. However, despite reaching readiness at discharge, feeding problems are sometimes underestimated and persist in childhood in this group of patients, which can have an important impact on the health of this population^(1)^.

Hence, there is a growing interest in studying oral, feeding, and neurodevelopment skills during early childhood, especially to understand which conditions may interfere in or predispose to difficulties in introducing consistencies during the period of complementary feeding in preterm infants.

Technological advances increasingly favor the survival of preterm newborns (PTNB) and, consequently, there is also an increase in comorbidities and developmental delays, including feeding difficulties^(1)^.

Although the ability to eat is a fine motor skill, global motor development is essential for an adequate oral function^(2)^. Thus, oral stability depends on head and shoulder control, which are related to torso and pelvis stability^(3)^.

A literature review study suggests that PTNB born with very low birth weight, when compared with full-term newborns (FNB), have more feeding difficulties that persist in the long term, during and beyond the introduction of complementary feeding^(4)^. Difficulties are already observed in the introduction of food and new consistencies, demonstrated through refusal, vomiting, crying, irritability, nausea, and frequent choking in this population^(5)^.

Although the literature reports such difficulties, there is still a scarcity of longitudinal studies that address the progression of food consistencies in the period of complementary feeding as well as the age at which complementary feeding started in the first year of life^(6)^. Therefore, understanding the factors that interfere in the process of oral motor development and the introduction of complementary feeding of PTNB can direct strategies and interventions in such a way that this population is monitored, even before presenting difficulties, improving the overall development of this population.

In this context, the objective of this study was to compare the introduction of consistencies during the introduction of complementary feeding between PTNB and FNB and to evaluate the presence of Oral Motor Dysfunction (OMD) in these groups, as well as to evaluate whether there is a correlation between OMD and difficulty in introducing consistencies.

## METHODS

This is an observational, analytical, cohort study, with ambispective data collection and approved by the Ethics Committee on Research Involving Human Beings of Universidade Federal do Paraná (UFPR), Department of Health Sciences, under Opinion No.: 2.439.032.

The study was carried out from October 2017 to November 2020, at the Outpatient Follow-up Service for Newborns at Risk (*Serviço Ambulatorial de Seguimento de Recém-nascido de Risco* - SAS-RNR) aimed at PTNB, conducted by the interprofessional team of the Expanded Centers for Family Health and Primary Care (*Núcleo Ampliado de Saúde da Família* - NASF-AB) composed of a speech therapist, a nutritionist, and a psychologist. The study was also developed in the Family Health Strategies (FHS) in which the FNB were evaluated by the same professionals. The three professionals participated and evaluated all newborns (NB) in the same consultation.

### Sample

The sample was selected in a non-probabilistic way, by convenience, but in a systematic way, with scheduled times, always on the same day of the week (Friday), in the same period of the day (morning - 7 am to 1 pm).

PTNB and those who were admitted in the Neonatal Intensive Care Unit (NICU) were referred by the maternity hospital to the interprofessional follow-up at the NASF-AB. Notably, during the period of PTNB hospitalization, the institution had only one speech therapist at the time of the study, making it unfeasible to work with all PTNB. FNB who had difficulty breastfeeding, observed or reported at the time of performing the heel prick test up to the fifth day of life in the FHS, were referred to evaluation by the same team.

The research included PTNB with gestational age (GA) ˂ 37 weeks and FNB with GA > 37 weeks at birth, whose mothers and/or guardians signed an Informed Consent Form. In addition, they should have attended all stages of the study, that is, the five proposed evaluations.

NB with any neurological or craniofacial alteration, and/or syndrome that interfered with normal orofacial and swallowing development, among other comorbidities, were excluded from both groups.

In addition, NB who had Grade III or Grade IV Peri-Intraventricular Hemorrhage and who had critical heart disease and/or clinical decompensation with medical diagnosis at any time during the study were excluded.

### Evaluation procedures and instruments

PTNB and FNB were submitted to the same evaluation protocols in the five consultations carried out during the follow-up proposed for this research: 1^st^ consultation - from 7 to 15 days after discharge; 2^nd^ consultation - at 4 months of life; 3^rd^ consultation - at 6 months; 4^th^ consultation - at 9 months; and 5^th^ consultation - at 12 months of life. Parents and/or guardians would leave the service with a scheduled appointment for follow-up and reassessment.

Data collection was carried out by the author with the participation of the nutritionist and the psychologist in the SAS-RNR of the municipality. Data collection was always carried out by the same professionals, previously trained to apply the study protocols. None of the instruments required a certificate for application.

In the first consultation, an interview was conducted with the mothers/guardians, by the researcher, using the Data Record Form standardized for this study, consisting of sociodemographic questions related to pregnancy, birth, data on hospitalization, and hospital discharge.

The anthropometric assessment was performed by the team’s nutritionist by measuring body weight, length, and head circumference. To measure body weight, a Balmak® digital pediatric scale was used, with a maximum capacity of 25 kg. To measure length, a wooden infantometer was used with a range of 10 to 99 cm with subdivisions of millimeters.

Prematurity, treated as an independent variable, was defined as gestational age < 37 weeks. It was also classified by the subcategories: Extreme Preterm (< 28 weeks), Very Preterm (28 to < 32 weeks), Moderate Preterm (32 to < 37 weeks), and Late Preterm (34 to < 37 weeks) according to the information recorded in the Child Health Handbook.

As dependent variables, the following were considered:

1) Oral motor dysfunction: defined as functional alteration of oral skills, caused by immature sucking pattern, incoordination between sucking/swallowing/breathing, difficulty chewing and swallowing, as well as oral inabilities in the use of different utensils^(2,7)^; this variable was obtained from the Adapted Protocol for the Assessment of Pediatric Dysphagia - PAD-PED^(8)^. Both in the PTNB and in the FNB, the Structural and Functional Examination of the orofacial sensorimotor system was performed. NB were positioned on a stretcher in the supine position with the head elevated, to check the oral and non-nutritive sucking reflexes. With a glove on, the little finger was used in the perioral region to stimulate the search reflex; subsequently, the anterior portion of the hard palate and the tip of the tongue were touched to elicit sucking.

Lip, tongue, and cheek mobility and tonus were evaluated under observation of posture during rest and mobility during performance of stomatognathic functions. This procedure was performed at all stages of follow-up, covering all stages of development.

2) Difficulty introducing food consistency: defined as oral motor alteration when faced with different food consistencies, different flavors and utensils^(9)^. For this evaluation, the same adapted PAD-PED Protocol^(8)^ was used based on the data obtained during the food offer:

For food offer, the following consistencies were used: thin liquid (breast milk/infant formula), thickened liquid (thickened milk), homogeneous pasty (mashed fruits/vegetables), heterogeneous pasty (small pieces of fruit/vegetables), and solid (fruits/vegetables in pieces); utensils were also used, depending on the age group. These foods were offered by the mother/caregiver in the usual feeding position, respecting what has been already introduced by the family.

Difficulties introducing consistencies were considered when, during the periods in which consistencies were offered, the NB still did not accept them at the time of the assessment and/or had oral motor difficulty with such consistency; this evaluation was performed by behavioral observation when the food was offered.

3) Assessment of neuropsychomotor development, performed using the Denver Developmental Screening Test II (DDST-II)^(10)^ and the Child Development Clinical Risk Indicators (IRDI)^(11)^.

Other interfering variables considered included the evaluation of breastfeeding observation, by the protocol disseminated by UNICEF^(12)^, in which it is possible to observe behaviors favorable to breastfeeding or suggestive of difficulties, considering the body position of the mother and the NB during breastfeeding, onset of breastfeeding, efficiency of sucking, affective involvement between mother and baby, anatomical characteristics of the breast, and duration and termination of breastfeeding. Based on the frequency of unfavorable behaviors for each aspect of the investigated breastfeeding, breastfeeding was classified as Good, Regular, or Bad^(13)^.

The mother’s emotional state was assessed as another possible interfering variable using the Edinburgh Postpartum Depression Scale (EPDS), which has already been translated into several languages with validation in several countries, including Brazil. This is a self-reported protocol that aims to identify and assess the intensity of postpartum depression symptoms, consisting of ten items that receive scores from zero to three according to the reported intensity of depressive symptoms^(14)^, and the total score ranges from 0 to 30, with scores equal to or greater than 12 being considered a sign of depression^(15)^.

Finally, low birth weight data were also considered, with weight < 2500 grams; and invasive oral procedures such as: presence and time of use of enteral nutrition; presence and time of use of mechanical ventilation; presence and time of use of Continuous Positive Airway Pressure (CPAP); and presence and time of use of oxygen tent or helmet to supply supplemental O2.

Data were collected and tabulated, exclusively by the researcher, by using a Microsoft Office Excel® spreadsheet (2013), and forwarded to a qualified professional for statistical analysis.

Continuous variables were evaluated regarding their distribution and are presented as arithmetic mean and standard deviation, for continuous variables with normal distribution, and median with interquartile range (25-75%) for those with asymmetrical distribution. Categorical variables are presented with their absolute and relative frequencies.

To estimate the difference between continuous variables, the Student’s t-test, Mann-Whitney test, and ANOVA were applied to repeated measures with Duncan’s *post-hoc* test.

To estimate the difference between categorical variables, Fisher’s exact test and Pearson’s chi-square test were applied.

The estimation of the difference between continuous variables with symmetrical distribution was performed using Student’s t-test and ANOVA for repeated measures with Duncan’s *post-hoc* test. For asymmetric variables, the Mann-Whitney test was performed. Categorical variables were evaluated using Fisher’s and Pearson’s chi-square tests.

The Multivariate Logistic Regression model was applied to identify the main factors associated with oral motor dysfunction. Magnitude of effect size of 25% for the main outcome, whichever the proportion of FNB and PTNB with oral motor dysfunction, type I error of 5% and type II error of 10%, were considered. The estimated sample was 44 cases in each group, considering a test power of 90% (*Statistica* v.10.0 - Statsoft®).

## RESULTS

### Characteristics of newborns and mothers

**Figure 1:**
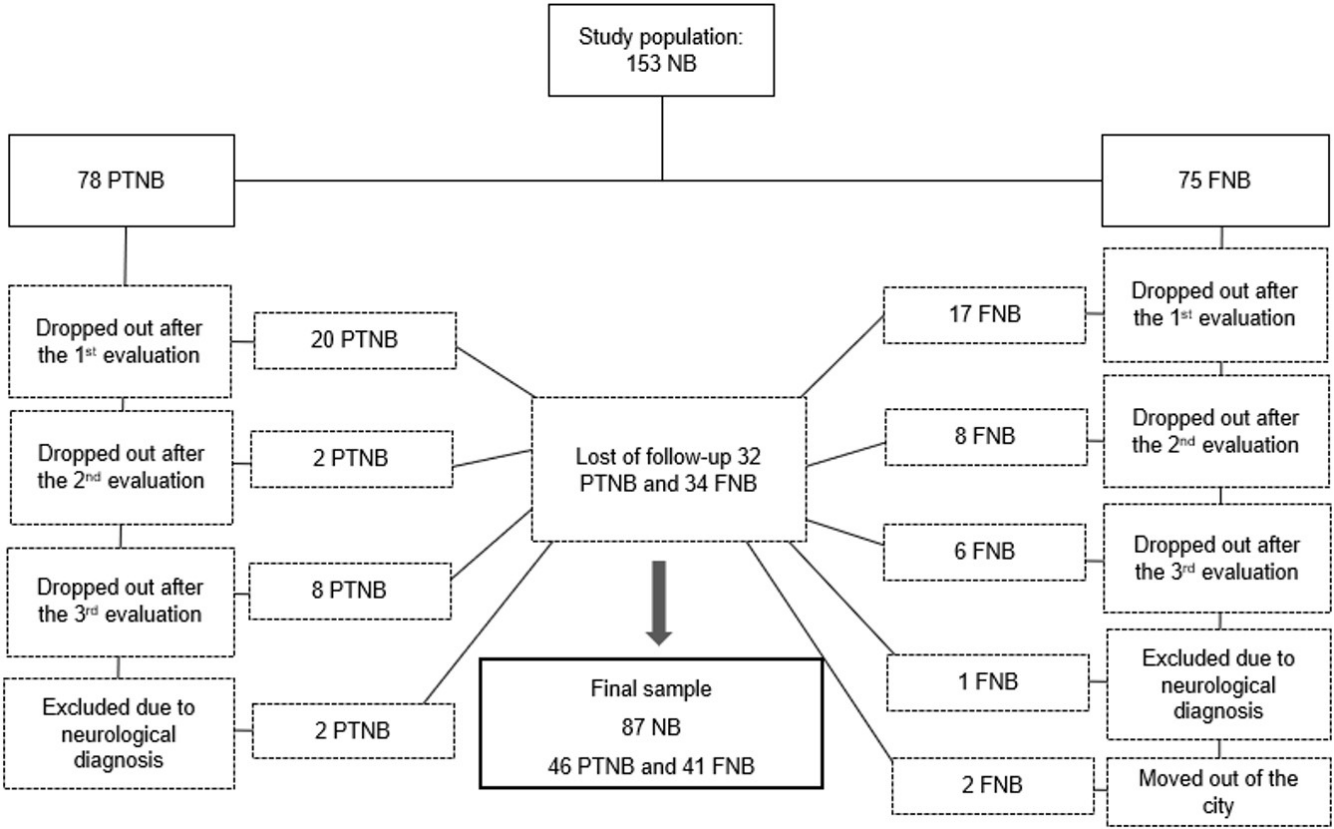
Study population

Source: Prepared by the author (2021)

During the study period, 153 NB who met the inclusion criteria were eligible. There were losses during the study due to dropout (n = 63) and due to the identification of a diagnosis of neurological alterations during follow-up (n = 3). The study sample consisted of 87 NB, of which 46 were in the PTNB group and 41 in the FNB group (Figure 1). In Table 1 we present the birth characteristics of the two NB groups.

**Table 1:** Birth characteristics of newborns - Municipal Department of Health (MAFRA-SC)

CHARACTERISTICS	FNB (n = 41)	PTNB (n = 46)	P
Sex			
Boy	18 (43.9%)	22 (47.8%)	0.82^2^
Girl	23 (56.1%)	24 (52.2%)	
Twinning	0 (0.0%)	12 (26.1%)	< 0.001^2^
Gestational age (weeks)	38.7 ± 1.0	34.1±2.3	< 0.001^3^
Birth weight (g)	3,230.0 ± 537.8	2,216.0 ± 570.2	< 0.001^3^
Gestational age and birth weight combined			
SGA	10 (24.4%)	16 (34.8%)	0.12^1^
AGA	28 (68.3%)	30 (65.2%)	
LGA	3 (7.3%)	0 (0.0%)	
Head circumference (cm)	34.0 ± 1.5	31.2 ± 2.5	< 0.001^3^
Length (cm)	47.7 ± 2.7	43.3 ± 3.1	< 0.001^3^
1^st^ minute Apgar score			
< 3	0 (0.0%)	*1 (2.2%)	0.42^1^
3-7	10 (24.4%)	15 (32.6%)	
> 7	31 (75.6%)	30 (65.2%)	
5^th^ minute Apgar score			
< 3	0 (0.0%)	0 (0.0%)	1.00^1^
3-7	3 (7.3%)	4 (8.7%)	
> 7	38 (92.7%)	42 (91.3%)	

1 Pearson’s chi-square test;

2 Fisher’s exact test;

3 Student’s t-test

* n = 1 ---: not applicable

Caption: SGA = Small for Gestational Age; AGA = Appropriate for Gestational Age; LGA = Large for Gestational Age; FNB = Full-term newborns; PTNB = Preterm newborns

In the PTNB group, we verified a higher frequency of twinning (26.1% *vs*. 0.0%, p < 0.001). The other characteristics—gestational age, birth weight, head circumference, and length—were evidently lower among PTNB (p < 0.001).

Nine FNB (21.9%) and 26 PTNB (56.5%) required hospitalization in the NICU, and their length of stay had a median of 6.0 (3-10) days and 13.5 (5-21) days, respectively. The main cause of hospitalization between PTNB was respiratory distress syndrome (18; 75.0%).

Among the 35 NB who required hospitalization in the NICU, all NB in both groups required an alternative feeding route; however, the time spent on enteral nutrition was, on average, significantly longer among PTNB (p = 0.03). Other invasive oral procedures were used in six FNB and 19 PTNB. Breast was the most used method of transition to the oral route in FNB; in PTNB, it was the use of a cup (p < 0.001); this is because it is a maternity that holds the title of Baby-Friendly Hospital Initiative, which uses this method as a way to carry out the transition and complement the diet of PTNB.

With regard to oxygen therapy, of the 9 FNB, 3 required mechanical ventilation; and, among the PTNB, 7 (26.9%). The length of stay had a median of 6 (4-10) days and 10 (2-35) days, respectively. We also observed a higher frequency of use of CPAP among PTNB (0% *vs.* 57.7%), with a significant difference, and the length of stay had a median of 3 (1-6) days.

No significant difference was observed in the type of feeding at hospital discharge between the two groups of NB, with 100% of both groups being breastfed, three NB and nine PTNB on mixed feeding.

We observed a higher frequency of previous maternal disease among PTNB (7.3% *vs.* 34.8%, p < 0.001), with the most frequent disease in mothers of FNB being arterial hypertension and in mothers of PTNB, depression. Regarding the history of maternal mental health, 8 (19.5%) of the mothers of FNB had a history of mental disorder, and 13 (28.3%) mothers of PTNB presented depression as their main mental disorder.

Table 2 shows the NB characteristics related to anthropometric data, such as length, head circumference, and weight, for all assessments.

**Table 2:** Chronological age, corrected age, weight, length, and head circumference - Municipal Department of Health (MAFRA-SC)

EVALUATIONS	FNB (n = 41)				
	Chronological age (days)	Corrected age (days)	Weight (g)	Length (cm)	Head circumference (cm)
1^st^ evaluation	27.0 (17.50)	NM	4,003.5 ± 834.8	53.4 ± 3.0	36.6 ± 2.0
2^nd^ evaluation	4.3 ± 0.3	NM	6,508.2 ± 740.4	62.0 ± 2.9	41.1 ± 1.5
3^rd^ evaluation	6.3 ± 0.4	NM	7,495.7 ± 881.2	65.4 ± 2.9	42.9 ± 1.2
4^th^ evaluation	9.4 ± 0.6	NM	8,916.2 ± 924.0	70.4 ± 2.7	45.0 ± 1.4
5^th^ evaluation	12.6 ± 0.4	NM	9,9917.6 ± 930.4	74.5 ± 2.2	46.3 ± 1.3
	PTNB (n = 46)				
	Chronological age (weeks)	Corrected age (weeks)	Weight (g)	Length (cm)	Head circumference (cm)
1^st^ evaluation	64.0 (53-83)	21.5 (16-29)	3,949.5 ± 877.7	52.0 ± 3.8	37.2 ± 2.5
2^nd^ evaluation	5.4 ± 0.9	4.0 ± 0.6	6,222.5 ± 962.2	60.3 ± 4.0	41.0 ± 1.9
3^rd^ evaluation	7.6 ± 0.9	6.2 ± 0.6	7,274.8 ± 1,022.1	64.4 ± 5.1	43.7 ± 3.1
4^th^ evaluation	10.6 ± 1.1	9.1 ± 0.8	8,347.8 ± 1,114.1	68.9 ± 4.8	44.5 ± 2.1
5^th^ evaluation	13.7 ± 1.0	12.2 ± 0.8	9,258.2 ± 1,185.8	73.4 ± 5.3	46.8 ± 4.0

Caption: FNB = Full-term newborns; PTNB = Preterm newborns; NM = not measured. Source: Prepared by the author (2022)

### Protocol for breastfeeding observation

Regarding the type of food offered to the NB, at hospital discharge there was a predominance of breast milk (BM) in both groups (p = 0.22). The frequency of feeding with BM was higher among the FNB in the 1^st^ evaluation (82.9% *vs.* 54.3%, p < 0.01). As of the third evaluation, there was a decrease in the frequency of offering BM in both groups, being observed 32.4% *vs*. 30% in the last evaluation.

The frequency of formula milk (FM) use was similar between groups (p > 0.05), while mixed feeding (BM + FM) was higher among PTNB in the 1^st^ evaluation (14.6% *vs*. 41.3%, p = 0.01).

During the breastfeeding assessment, in the 1^st^ evaluation, we observed a significant difference only in relation to sucking between FNB and PTNB (p < 0.01), with a higher frequency of difficulty in PTNB (27.5% *vs.* 4.5*%*). In the other evaluations, in all assessed items, all NB in both groups presented a good classification, according to the applied protocol (p = 1.00). Difficulty breastfeeding was observed in 17 FNB (41.5%) and 15 PTNB (34.1%) (p = 0.50) without association with the type of feeding in the 1^st^ evaluation (p = 0.87).

### Feeding assessment

#### Feeding history

In both groups, we verified a change from lying down to sitting position between the 2^nd^ and 3^rd^ evaluations (p < 0.001). The duration of breastfeeding was significantly longer among the FNB in the 1^st^ evaluation (p < .01), with no difference in the other evaluations (p > 0.05).

**Figure 2:**
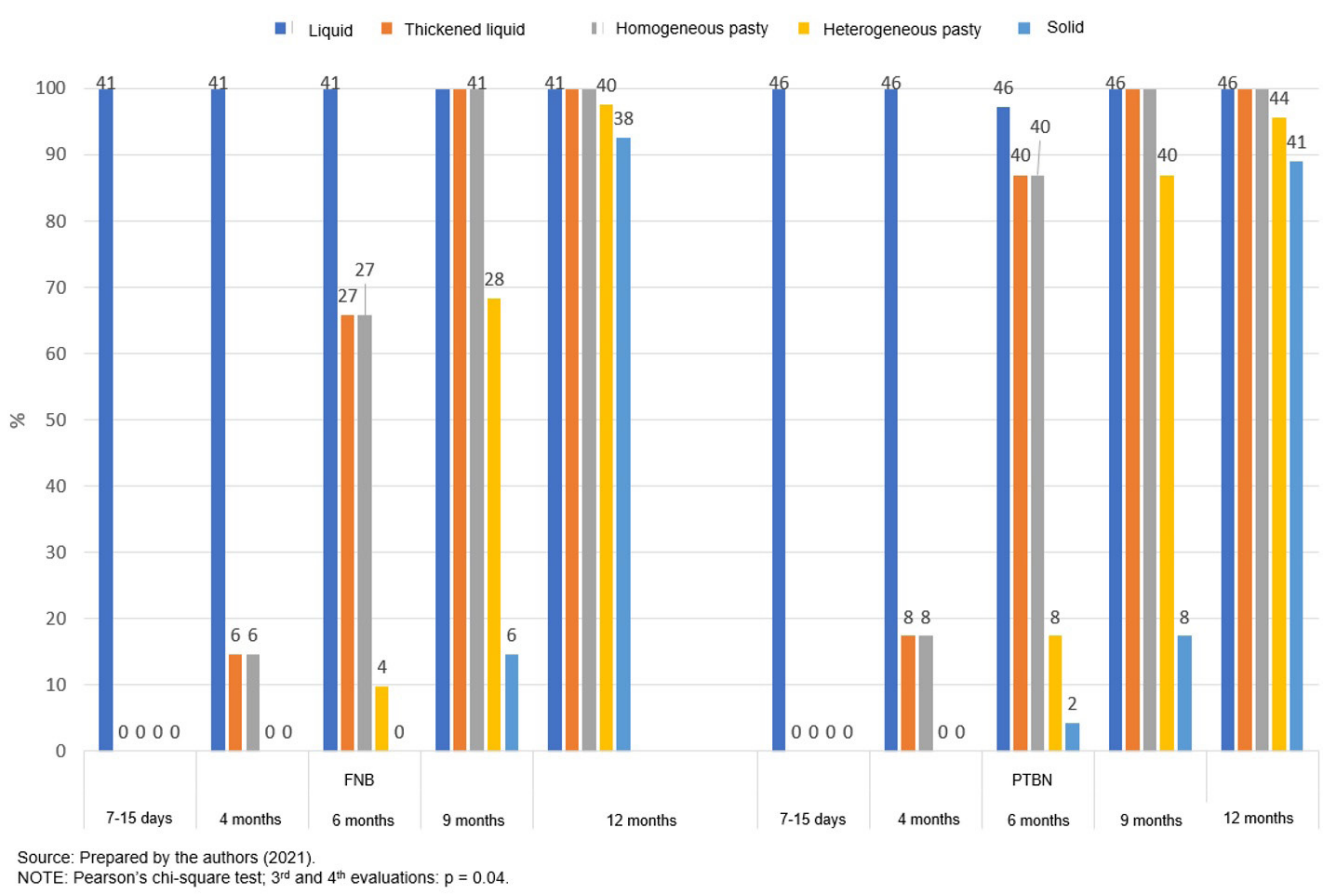
Introduction of food consistencies - Municipal Department of Health (MAFRA-SC)

In Figure 2 we illustrate the introduction of consistencies observed in the two groups of NB, that is, which consistencies the NB accepted during the study period. In the 3^rd^ evaluation, a higher frequency of administration of thickened liquid consistency (in this case, NB exposed to liquids such as thickened milk) and homogeneous pasty consistency was observed for PTNB (84.8% *vs*. 65.8%, p = 0.04). In the 4^th^ evaluation, the administration of a heterogeneous pasty consistency was more frequent, also among PTNB (97.0% *vs.* 68.3%, p = 0.04).

The use of a bottle as a feeding utensil was more frequently used among PTNB in the 1^st^ evaluation (17.1% *vs.* 45.6%, p < 0.001) and 2^nd^ evaluation (51.2% *vs.* 73.9%, p < 0.01); and the use of spoons and cups, among FNB in the 3^rd^ evaluation (90.2% *vs*. 73.9%, p = 0.02) and 4^th^ evaluation (100% *vs.* 84.8%, p = 0.02).

Table 3 shows the ages at which each consistency was offered for the first time to the NB (for PTNB, corrected age (CA) was considered), as well as the duration of exclusive breastfeeding (EB) and weaning, with no significant difference being observed.

**Table 3:** Age of nb regarding: exclusive breastfeeding; weaning; onset of complementary feeding; and age at introduction of different food consistencies - Municipal Department of Health (MAFRA-SC)

AGE (DAYS)	FNB (n = 41)	PTNB (n = 46)	P
Exclusive breastfeeding	122.0 (62-183)	122.0 (30-152)	0.04^1^
Weaning	250.0 (150-365)	239.5 (143.5-365)	0.65^1^
Onset of complementary feeding	169.9 ± 22.7	169.9 ± 22.7	0.24^2^
Introduction of homogeneous pasty consistency	169.9 ± 22.7	169.9 ± 22.7	0.24^2^
Introduction of heterogeneous pasty consistency	259.6 ± 53.7	247.6 ± 51.7	0.49^2^
Introduction of solid foods	323.1 ± 39.5	321.8 ± 51.6	0.90^2^

1 Mann-Whitney test;

2 Student’s t-test

Caption: FNB = Full-term newborns; PTNB = Preterm newborns. Source: Prepared by the author (2021)

In the case of EB, although the medians are the same, the variation around the median is different between groups. In the statistical analysis, considering dispersion, we verified p = 0.04, indicating that there is a difference, but not considerable, and therefore the p-value is close to 0.05.

#### Structural and functional examination

In the structural and functional examination of the lips and tongue, we found no difference between the groups (p > 0.05). The posture of parted lips was observed in 37 (90.2%) of the FNB and in 37 (80.4%) of the PTNB; and the posture of tongue on papilla, in 34 (82.9%) *vs.*37 (80.4%), respectively. Decreased tongue tonus was observed in one (2.4%) *vs.* six (13.0%).

In the assessment of non-nutritive sucking, in both groups, a search reflex was observed in 29 (70.7%) of the FNB and 25 (54.3%) of the PTNB. We observed no significant difference in the sucking pattern between the FNB and PTNB groups, with 32 (78.1%) and 41 (89.1%) being adequate. Intraoral pressure was adequate in 32 (78.1%) of the FNB and 41 (89.1%) of the PTNB.

#### Functional assessment with food

In the assessment with food, in relation to the mother’s breast, a better lip tie was observed among the FNB (67.5% *vs.* 32.5%, p = 0.03) and better latching among the PTNB (93.2% *vs.* 67 .5%, p < 0.001). In the ratio between sucking/swallowing frequency and coordination, there was no significant difference.

In the thin liquid consistency, we verified no difference between the groups in the items: lip tie, oral escape, coordination, and feeding time (p > 0.05).

Considering the variables: coordination, latch, lip tie, leaking through the labial commissure, and inadequate sucking, we observed OMD in 15 cases (36.6% *vs.* 32.6%) in each group in the 1^st^ evaluation. Among the NB with OMD, there was persistence in two cases among the FNB and three cases among the PTNB in the last evaluation; in the latter, we observed difficulty in preparing and chewing for heterogeneous pasty and solid consistencies, open-cup anterior leakage of liquid. In Table 4 we present the main characteristics of the five NB who presented with OMD in the last evaluation.

**Table 4:** Main characteristics of newborns with oral motor dysfunction in the last evaluation - Municipal Department of Health (MAFRA-SC)

N	GROUP	APGAR	CD	IOP	TI	BHD	BF DF	POOR SUCKING	OMD 7-15 DAYS	DENVER	PSYCHOLOGICAL RISK	IRDI
1	PTBN	9/10	Yes	Yes	No	No	Yes	Yes	Yes	Normal	No	Altered
2	PTBN	7/9	Yes	Yes	Yes	Yes	No	No	Yes	Normal	No	Normal
3	PTBN	4/7	Yes	Yes	No	No	Yes	No	Yes	Normal	No	Normal
4	FNB	9/10	Yes	Yes	Yes	No	Yes	No	Yes	Abnormal	No	Normal
5	FNB	8/8	No	No	No	No	No	No	No	Normal	No	Normal

Caption: FNB = Full-term newborns; PTNB = Preterm newborns; APGAR = APGAR index, is a test done on the newborn shortly after birth that assesses its general condition and vitality; CD = Cardiopulmonary diagnosis; IOP = Invasive oral procedure; TI = Tracheal intubation; BHD = Bottle at hospital discharge; BF DF = Breastfeeding difficulty; OMD = Oral Motor Dysfunction; IRDI = Child Development Clinical Risk Indicators Altered: two absences or more. Source: Prepared by the author (2021)

Among the main differences between FNB and PTNB with OMD are twinning (0% *vs.* 33.3%), NICU hospitalization (33.3% *vs.* 80%), use of CPAP (0% *vs.* 46.7%), and nasogastric tube (13.3% *vs*. 73.3%). In addition, FNB more frequently presented difficulty breastfeeding and poor sucking (p < 0.01), with a significant difference in the latter.

Considering the analysis of all without OMD (n = 57) and with OMD (n = 30), we observed that there was no difference in relation to the frequency of prematurity (p = 0.82) and neuropsychomotor development abnormalities (p = 0.20) between groups. The variables associated with OMD were: difficulty breastfeeding, response to breastfeeding, poor sucking, NICU hospitalization, invasive oral procedures, and use of a bottle at hospital discharge (Table 5).

**Table 5:** Main differences of newborns with oral motor dysfunction in the 1^st^ evaluation - Municipal Department of Health (MAFRA-SC)

CHARACTERISTICS	WITHOUT OMD (n = 7)	WITH OMD (n = 30)	p
Gestational age	36.5 ± 2.4	35.9 ± 3.7	0.34^1^
Preterm	31 (54.4%)	15 (50.0%)	0.82^2^
Birth weight	2728.5 ± 672.0	2628.0 ± 890.5	0.55^1^
ICU Hospitalization	18 (31.6%)	17 (56.7%)	0.03^2^
Tracheal intubation	2 (3.6%)	8 (26.7%)	0.02^2^
Invasive oral procedures	18 (31.6%)	17 (56.7%)	0.03^2^
Difficulty breastfeeding	15 (26.3%)	17 (56.7%)	< 0.01^2^
Response	2 (3.6%)	9 (30.0%)	< 0.001^2^
Sucking	3 (5.4%)	14 (46.7%)	< 0.001^2^
Bottle at hospital discharge	3 (5.4%)	9 (30.0%)	< 0.01^2^
Denver Abnormality	8 (14.0%)	7 (23.3%)	0.20^2^

1 Student’s t-test;

2 Fisher’s exact test

Caption: OMD = Oral Motor Dysfunction; ICU = Intensive Care Unit. Source: Prepared by the author (2021)

In the analysis of the main predictive factors for OMD, using multivariate logistic regression, bottle feeding increased the odds of OMD by approximately seven times (OR = 7.55; 95%CI: 1.66-34.18; p < 0.01) and the occurrence of invasive oral procedures by about five times (OR = 4.95; 95%CI: 1.09-22.27; p = 0.02); we did not observe the same relation to the indicators: NICU hospitalization, difficulty breastfeeding, and sucking.

### Neurodevelopmental assessment

In the assessment of development using the Denver test, we observed a higher frequency of cases classified as abnormal among PTNB in the 2^nd^ and 3^rd^ evaluations.

Among the items evaluated in the protocol in the personal-social component, a higher frequency of suspected and abnormal classification was observed in the 1^st^ evaluation among PTNB (suspected: 15.2% *vs*. 2.4%; abnormal: 8.7% *vs.* 2.4%, p = 0.04). We verified no significant difference in language classification between groups in any of the evaluations (p > 0.05).

In the fine motor component, we observed no difference between groups (p > 0.05). For the gross motor component in the 2^nd^ evaluation (suspected: 30.4% *vs.* 12.2%; abnormal: 21.7% *vs.* 7.3%, p < 0.001) and 3^rd^ evaluation (suspect: 10.9% *vs.* 2.4%; abnormal: 28.3% *vs.* 2.2%, p = 0.03), there was a higher frequency of suspicious and abnormal cases between PTNB.

When associating the records of the Denver II protocol with the OMD assessment, we observed that, in the 1^st^ evaluation, in three cases there was a delay in Denver in the Gross Motor item, one of them with OMD; in the 2^nd^ evaluation, 18 cases, none with OMD; in the 3^rd^ evaluation, 13 cases, none with OMD; and in the 4^th^ evaluation, seven cases, one with OMD, with no difference between FNB and PTNB; there was no evidence of association between gross motor dysfunction and OMD (p > 0.05).

### Child Development Clinical Risk Indicators (IRDI)

We verified no significant difference between risk indicators for child development in all assessments (p > 0.05). However, we identified psychological risk at 2.2% only for the PTNB group (p = 1.00).

### Screening the mother’s emotional state

We used the Edinburgh Postpartum Depression Scale to screen the mother’s emotional state, with the presence of signs of depression in 11 cases in the FNB group (26.8%) and seven cases in the PTNB group (15.2%) (p = 0.19) in the 1^st^ evaluation. In the 2^nd^ evaluation, these frequencies decreased to 7.3% (three cases) and 2.2% (one case) (p = 0.33), all of whom were referred to psychological guidance and there were no cases or persistence of symptoms in the following evaluations.

## DISCUSSION

This study sought to assess the presence of OMD in PTNB and compare it with FNB, as well as to assess whether there is a correlation between OMD and difficulty introducing food consistencies in these populations; and our main result was that, although we observed OMD, there was no difference between PTNB and FNB. Moreover, despite the occurrence of OMD, there was no difficulty in starting complementary feeding, which occurred early. OMD was observed in 15 cases, in each group (FNB and PTNB, respectively) in the first evaluation and there was persistence of OMD in two cases of FNB and three cases among PTNB in the last evaluation. In the latter, we observed difficulty in preparing and chewing for heterogeneous pasty and solid consistencies, corresponding to the difficulty for these consistencies and open-cup anterior leakage of liquid, corresponding to oral difficulty with this utensil.

The actual prevalence of swallowing problems and OMD in neonates and infants is unknown. Studies have shown that in the assessment of PTNB at four months, the presence of OMD ranged from 23% to 89% for pasty consistency; at six months, they identified OMD in approximately 40% of the PTNB in the sample for the semi-solid consistency; and at 12 months, a variation between 8% and 28% for the solid consistency^(6,16-18)^.

In these studies, GA at birth was 32 weeks on average, whereas in the present study it was 34 weeks. It is noteworthy that this difference of two weeks of GA translates into a different neurophysiological evolution, i.e., there is a great difference in neurological maturity and, consequently, different levels of oral motor skills are observed.

In our study, OMD was not associated with delays in neuropsychomotor development assessed by the Denver II, but high neurological risk patients were excluded from the sample. Moreover, the patients were followed up by an interdisciplinary team, and the necessary guidelines regarding food and nutrition, in addition to psychosocial interventions, were carried out at each evaluation. Therefore, a risk of intervention bias may have been responsible for the low frequency of alterations in this study. In addition, the population of this study was predominantly of late PTNB and not of extreme preterm infants, the latter being more predisposed to the risk of OMD than the former.

The prevalence of feeding problems described in a population study in the United Kingdom in 2001, with 14 thousand preterm infants born at < 37 weeks of gestation, was 10.5%, and this frequency increased to 24.5% among those born with very low birth weight (< 1500 g)^(19)^. This study also relates persistent dietary changes in the first 15 months of life to delay in neuropsychomotor development. Conversely, feeding difficulties in the first four weeks of life are very common and do not have an important predictive value.

In the analysis of the 30 NB (15 in both groups) with OMD, we found no association with prematurity and neuropsychomotor development, but it was possible to verify the association with difficulty breastfeeding, NICU hospitalization, neonatal oral invasive procedures, and bottle feeding at hospital discharge.

Studies indicate that PTNB are exposed to prolonged and harmful external stimuli, such as endotracheal tubes and orogastric tubes, and that such interventions can negatively impact the oral skills of this population^(1,20,21)^, with a potential risk of aversion to oral feeding in the medium and long term^(1,17)^.

Regarding the five NB with OMD in the last evaluation, four underwent invasive oral interventions and already had OMD in the first assessment. When performing the analysis of predictors for OMD, the occurrence of invasive oral procedures increased the odds of presenting such difficulty by six times.

In a population study in the United Kingdom, using a questionnaire applied by telephone, comparing 1,130 PTNB with 1,255 FNB, PTNB had more feeding difficulties at two years of age. The relative risk of feeding difficulties was 1.57 and 1.62 for OMD, and the use of a nasogastric tube for more than two weeks was associated with feeding difficulties^(22)^.

In another Brazilian cross-sectional study on 62 PTNB, time spent using an enteral tube was also associated with feeding difficulties and defensive behaviors at 13 months of corrected age. However, no association was found between OMD and feeding difficulties^(6)^.

Two other studies report a significant association between eating difficulties and GA; the study population was extreme PTNB^(5,6)^. Another cross-sectional study, also on PTNB with an average GA of 32 weeks, did not find a relation between OMD and GA^(16)^, as in our study. As aforementioned, the population of this study was late PTNB, which may explain the lack of association with GA.

The period of introduction of complementary feeding, as well as the appropriate age for starting oral feeding with exposure to textures and flavors, respecting the windows of opportunity and all the stimuli and experiences that involve the relationship with food and the development of oral and motor skills, may be involved in feeding difficulties in PTNB in the medium and long term^(2)^.

Our results demonstrated that both in the FNB and in the PTNB, the introduction of the pasty consistency occurred early, around four months of life, corroborating studies carried out with preterm infants, in which they were exposed to the offer of fruits/porridges before completing six months of corrected age^(5,6,18,20,22-25)^.

The heterogeneous pasty consistency (small pieces) was observed more frequently in the 4^th^ evaluation, in which the NB had an average age of eight months (PTNB corrected age). Conversely, solid consistency started in both groups at around ten months, as expected for age. Nevertheless, we observed a small portion of NB that, in the 5^th^ evaluation, still did not accept either small pieces or solids, being considered a delay in their introduction.

When addressing the introduction of consistencies, the literature uses the ages of windows of opportunity as bases. At the sixth month of corrected age, complementary feeding is introduced, which should occur gradually in a pasty consistency; at eight months, the infant is already able to receive food in small pieces and/or shredded. This should not last longer than nine months, which could cause feeding problems in the future, and the introduction of a solid consistency similar to that of the family should be carried out up to 12 months^(6,9)^.

In addition, King (2009) stresses that the introduction of complementary feeding must respect the skills and pace of PTNB, so that they develop the appropriate skills for each progression of texture.

The offer of food, water, teas, and juices before six months of life already characterizes the early introduction of complementary feeding^(25)^. Both the Brazilian Ministry of Health and the Brazilian Society of Pediatrics do not recommend this practice, as it may lead to a decrease in exclusive breastfeeding or even weaning^(26,27)^.

Despite the recommendation of the Ministry of Health (2010) that breast milk should be exclusive until six months of life for the PTNB population. The literature is still scarce and there is no consensus; however, there is a recommendation for the introduction of complementary feeding to start as of six months of corrected age, and the signs of readiness must also be present^(28)^.

Notably, when complementary feeding is introduced early, the child may develop allergic diseases or even alterations in oral development, resulting in chewing difficulties. Conversely, when it occurs late, there may be a growth deficit or anemia, compromising the growth and development of facial structures^(5)^.

With regard to the average length of stay at the NICU, the length of stay depends on the complexity and degree of prematurity of the patients treated at the service. Most studies with highly complex NICUs describe mean length of stay for PTNB and those with low birth weight of over one month^(29)^. In one study, of the NBs that required admission to the NICU, 18 were FNB (31.6%) and 17 were PTNB (56.7%). The average length of stay at the NICU was 6 (3-10) days in the FNB group and 13.5 (5-21) days in the PTNB group, the latter being more susceptible to peri- and postnatal complications, thus requiring intensive care^(6)^. This data demonstrates that PTNB from the maternity hospital involved in the service were not highly complex patients. It also shows that many newborns in the studied group, instead of being discharged after 48 to 72 hours, remained hospitalized for a longer time, which indicates that the studied FNB population, although not having the risk of prematurity, had other risk factors, for example, admission to the NICU.

An important sampling bias to report was that the FNB were also considered at risk, in addition to some requiring NICU hospitalization. Others were selected based on difficulties in breastfeeding during the heel prick test and were referred to the NASF for specialized evaluation. Anyhow, OMD was also observed in the FNB, which indicates that even in this group with a theoretically favorable prognosis, according to the gestational age, the risk exists, and a specialized evaluation and screening should be considered, mainly in those more exposed to NICU admission.

In our study, 9 (21.9%) FNB were hospitalized in the NICU, with an average time of 6.0 (3-10) days remaining. Most of them had a cardiopulmonary diagnosis as the cause of hospitalization. Although no alterations were observed in the Development Screening, it must be considered that such a diagnosis can lead to delays in fine motor development.

At hospital discharge, the frequency of breastfeeding was high: 100% in FNB and 97% in PTNB, mixed in 7.3% and 19.6%, respectively. The Baby-Friendly Hospital Initiative has played a crucial role in mobilizing the actors involved within hospital institutions, in the process of changing behaviors and routines in view of the high rates of early weaning^(21)^.

The Maternidade Dona Catarina Kuss is an institution that is part of the Baby-Friendly Hospital Initiative and has the Kangaroo Mother Care method, both of which provide, encourage, and promote breastfeeding^(25)^. Mothers of PTNB remain hospitalized along with their children and are qualified and trained for breastfeeding, by assistance, strategies, and interventions that promote it effectively and safely before discharge.

Although there are the Breastfeeding Strategy and Feeding Brazil Strategy, which are actions to strengthen the promotion, protection, and support of breastfeeding and healthy complementary feeding for children under two years of age^(21)^, the municipality’s numbers are much lower than expected; between hospital discharge and the 1^st^ evaluation, we observed a decrease in the frequency of breastfeeding in both groups. However, this frequency was even lower in PTNB, becoming similar in the other evaluations. It is necessary to formulate strategies that can narrow the assistance to protect breastfeeding.

With regard to the characteristics of the assessment of breastfeeding, we observed a significant difference in the sucking item, with a higher frequency of difficulty in FNB. A better lip tie was observed in the FNB and a better latch in the PTNB. A hypothesis for this datum would be due to the fact that FNB were selected for this study based on the request for evaluation and management of breastfeeding, that is, they presented some degree of difficulty or complaint during breastfeeding, while PTNB already came from the maternity with these characteristics better established. This datum emphasizes that difficulties with breastfeeding do not occur only in the PTNB population; on the contrary, perhaps some FNB needed more time and more professional support to establish breastfeeding, as FNB exposed to risk factors for the development also deserve to receive specialized evaluation and follow-up.

It should be noted that breast milk is the best and most complete food for newborns, whether they are preterm or full-term, and that after the introduction of complementary feeding, breastfeeding is recommended for up to two years of life or more^(25)^. The time of exclusive breastfeeding was below the recommended (180 days, six months), in both groups with a mean age of 122 days (four months); and the mean age of weaning was eight months in the FNB and seven months of corrected age in PTNB.

In addition to organic, emotional, and environmental issues, when it comes to exclusive breastfeeding (EB), it must be considered that, currently, the Brazilian Labor Laws Consolidation do not corroborate the recommendations of the World Health Organization (WHO), with a four-month maternity leave. This, in addition to the little or limited support network, institutions/day-care centers that do not support the supply of breast milk, social vulnerability, among others, favors low EB rates.

In the results of the Child Development Clinical Risk Indicators (IRDI), despite some absences of two or more items being observed, most indicators were present; when absent, interprofessional intervention was carried out and the indicators were reassessed in the next consultation. Furthermore, it is worth noting that to be considered a psychological risk, the absent indicators must persist in the 2^nd^ evaluation. Such results may be related to the very characteristics of Maternidade Dona Catarina Kuss, the maternity from which the NB came, in addition to the fact that they are all inserted in a Follow-up Service for NB at Risk regarding development directed, among other aspects, towards the promotion of maternal-infant mental health.

As for the sample of this study, the proposed prolonged follow-up was affected by several variables that culminated in a significant loss of follow-up. Concerned about the loss of follow-up in prospective cohort studies in early childhood, as this is an issue faced worldwide, Keys et al.^(30)^ are conducting a systematic review research in Canada with the aim of surveying the elements which lead to failures in the recruitment and retention of parents in studies and follow-up centers for children aged 0 to 36 months. At the end of the research, they aim to offer recommendations for future research to adopt more efficient strategies for recruiting and, especially, for retaining participants in this population. Overall, studies with larger samples are relevant for the evaluation of the variables described in this study, ideally with healthy FNB without intervention and with a large population of extreme PTNB. For the true prevalence of OMD, studies preferably without intervention bias are needed. Nonetheless, as the assessment of OMD is carried out by specialists in speech therapy, an intervention-free assessment would be contrary to an adequate conduct.

## CONCLUSION

We observed no difference in the introduction of consistencies in the period of onset of complementary feeding between FNB and PTNB. Pasty consistency was introduced early in both groups.

Difficulties in breastfeeding occurred only in the first evaluation and in a small proportion, with no difference between FNB and PTNB.

The frequency of breastfeeding was higher among the FNB in the first evaluation, being similar as of the other evaluations; however, both EB and weaning are far below what is recommended in both groups.

OMD occurred in a small portion of both groups in the first evaluation (PTNB and FNB, respectively) and there was no persistence of difficulty in the different consistencies in the period of onset of food introduction. In the last evaluation, we observed OMD at a lower frequency than in the first, with a delay in the introduction of solids being observed in some cases.

With regard to OMD predictors, we verified that the bottle increased the odds of OMD by about seven times, and invasive oral procedures by about six times.

We observed no association between Neuropsychomotor Development, OMD and breastfeeding.

We verified no association between maternal depression and breastfeeding.
