# Supplementary material for: Comparison of the introduction of consistencies in complementary feeding introduction between preterm and full-term newborns - Cohort from 0 to 12 months
Source: Codas. 2023 Oct 13;36(1):e20220315. doi: 10.1590/2317-1782/20232022315en (PMC10715579; doi:10.1590/2317-1782/20232022315en)
Supplement: Translation

# Translation: Comparação da introdução das consistências na introdução da alimentação complementar entre recém-nascidos prematuros e a termo - Coorte de 0 a 12 meses

**DOI:** 10.1590/2317-1782/20232022315pt

**Authors:** Hellen Nataly Correia Lagos Guimarães, Andriéllen Marciniak, Lívia dos Santos Paula, Sheila Tamanini de Almeida, Adriane Celli

**Affiliations:** 1 Programa de Pós-graduação em Saúde da Criança e do Adolescente, Universidade Federal do Paraná - UFPR - Curitiba (PR), Brasil.; 2 Departamento de Fonoaudiologia, Universidade Federal de Ciências da Saúde de Porto Alegre - UFCSPA - Porto Alegre (RS), Brasil.

**Keywords:** Comportamento Alimentar, Alimentação Complementar, Aleitamento Materno, Crescimento, Recém-Nascido Prematuro

## Abstract

**Objetivo:**

Comparar a introdução das consistências no período da alimentação complementar de recém-nascidos prematuros e recém-nascidos a termo até os 12 meses de vida, bem como avaliar a presença de disfunção motora oral e a sua relação com dificuldade na introdução das consistências alimentares nestes grupos.

**Método:**

Trata-se de um estudo do tipo observacional, analítico, coorte, com coleta de dados ambispectiva, realizado na Secretaria Municipal de Saúde de Mafra - SC. A amostra do estudo foi constituída de 87 recém-nascidos, sendo 41 a termo e 46 prematuros. Na coleta de dados, foram realizadas entrevistas com as mães/responsáveis. A avaliação antropométrica foi realizada por nutricionista por meio de mensuração do peso corporal, comprimento e perímetro cefálico, seguida de avaliação das habilidades motoras orais e funcionais por meio do PAD-PED adaptado, avaliação da mamada, avaliação do desenvolvimento neuropsicomotor, avaliação da presença de depressão materna e risco psíquico da criança, até os 12 meses de vida de idade corrigida.

**Resultados:**

A disfunção motora oral foi observada em 15 recém-nascidos, em ambos os grupos, na consistência líquida na primeira avaliação, persistiu em dois casos nos recém-nascidos a termo e em três casos nos recém-nascidos pré-termo, na última avaliação para a consistência sólida.

**Conclusão:**

Não foi observada diferença na introdução das consistências alimentares entre os grupos. O aleitamento materno foi mais frequente nos a termo na primeira avaliação e semelhante nas demais avaliações. Com relação aos preditivos para disfunção motora oral, a mamadeira aumentou a chance em cerca de 7 vezes e procedimentos orais invasivos cerca de 6 vezes.

## INTRODUÇÃO

A competência para alimentação oral em prematuros é considerada um requisito essencial para a alta hospitalar. Porém, apesar de atingir a prontidão na alta, problemas de alimentação, por vezes, são subestimados e persistem na infância neste grupo de pacientes, o que pode gerar um impacto importante na saúde desta população^(1)^.

Desta maneira, é crescente o interesse em estudar as habilidades orais, alimentares e do neurodesenvolvimento durante a primeira infância, sobretudo entender quais condições podem interferir ou predispor a dificuldades na introdução das consistências durante o período de alimentação complementar em prematuros.

Os avanços tecnológicos cada vez mais favorecem a sobrevida dos recém-nascidos pré-termo (RNPT), e consequentemente, há também um aumento nas comorbidades e atrasos do desenvolvimento, incluindo as dificuldades alimentares^(1)^.

Embora a habilidade do comer se trate de uma habilidade motora fina, o desenvolvimento motor global é essencial para uma adequada função oral^(2)^. Assim a estabilidade oral depende do controle de cabeça e ombros, os quais se relacionam com a estabilidade de tronco e pelve^(3)^.

Um estudo de revisão de literatura sugere que RNPT nascidos com muito baixo peso, quando comparados aos recém nascidos a termo (RNT), apresentam mais dificuldades alimentares que persistem em longo prazo, durante e além da introdução da alimentação complementar^(4)^. Observam-se dificuldades já na introdução alimentar e na introdução de novas consistências, demonstradas por meio de recusa, vômito, choro, irritabilidade, náuseas e engasgos frequentes nesta população^(5)^.

Embora a literatura relate tais dificuldades, ainda há uma escassez de estudos longitudinais que abordem a progressão das consistências alimentares no período de alimentação complementar, bem como a idade de início destas no primeiro ano de vida^(6)^. Portanto, entender os fatores que interferem no processo de desenvolvimento motor oral e de introdução da alimentação complementar de RNPT pode direcionar estratégias e intervenções para que esta população seja acompanhada, mesmo antes de apresentar dificuldades, melhorando o desenvolvimento global desta população.

Neste contexto, o objetivo deste estudo foi comparar a introdução das consistências durante a introdução da alimentação complementar entre RNPT e RNT e avaliar a presença de Disfunção Motora Oral (DMO) nestes grupos, bem como avaliar se há correlação entre DMO e dificuldade na introdução das consistências.

## MÉTODO

Trata-se de um estudo observacional, analítico, coorte, com coleta de dados ambispectiva e aprovado pelo Comitê de Ética em Pesquisas em Seres Humanos, da Universidade Federal do Paraná - UFPR - Setor de Ciências da Saúde, Parecer Nº: 2.439.032.

Foi realizado no período de outubro de 2017 a novembro de 2020, no Serviço Ambulatorial de Seguimento de Recém-nascido de Risco (SAS-RNR) destinado aos RNPT, conduzido pela equipe interprofissional do Núcleo Ampliado de Saúde da Família (Nasf-AB) composta por fonoaudióloga, nutricionista e psicóloga. O estudo, também, foi desenvolvido nas Estratégias de Saúde da Família (ESF) em que os RNT foram avaliados pelas mesmas profissionais. As três profissionais participaram e avaliaram na mesma consulta todos os recém-nascidos (RN).

### Amostra

A amostra foi selecionada de forma não probabilística, por conveniência, mas de forma sistematizada, com horários agendados, sempre no mesmo dia da semana (sexta-feira), no mesmo período do dia (manhã - 7h às 13h).

Os RNPT e aqueles que passaram pela Unidade de Terapia Intensiva Neonatal (UTIN) foram encaminhados pela maternidade para seguimento interprofissional no NASF-AB. Vale ressaltar que durante o período de internação dos RNPT, a instituição contava na época do estudo, somente com uma fonoaudióloga, tornando-se inviável o trabalho com todos os RNPT. E os RNT que apresentaram dificuldade no aleitamento materno, observada ou relatada no momento da realização do teste do pezinho até o quinto dia de vida na ESF foram encaminhados para avaliação pela mesma equipe.

Foram incluídos na pesquisa, RNPT com idade gestacional (IG) ˂ de 37 semanas e RNT, com IG > de 37 semanas ao nascimento, para os quais as mães e/ou responsáveis assinaram o Termo de Consentimento Livre Esclarecido - TCLE. Além disso, deveriam ter comparecido a todas as etapas do estudo, ou seja, nas cinco avaliações propostas.

Foram excluídos RN com qualquer alteração neurológica, craniofacial e/ou síndrome que interfira no desenvolvimento normal orofacial e de deglutição entre outras comorbidades nos dois grupos.

Também, foram excluídos RN que apresentaram Hemorragia Peri-Intraventricular Grau III ou Grau IV e que apresentaram cardiopatia crítica e/ou com descompensação clínica com diagnóstico médico em qualquer momento do estudo.

### Procedimentos e instrumentos de avaliação

Os RNPT e RNT foram submetidos aos mesmos protocolos avaliativos nos cinco atendimentos realizados durante o acompanhamento proposto para esta pesquisa: 1° atendimento - de 7 a 15 dias após a alta; 2° atendimento - com 4 meses de vida; 3° atendimento - com 6 meses; 4° atendimento com 9 meses e 5° atendimento - com 12 meses de vida. Os pais e/ou responsáveis saiam do atendimento com retorno agendado para acompanhamento e reavaliação.

A coleta de dados foi realizada pela autora com coparticipação da nutricionista e da psicóloga no SAS-RNR do município. Esta foi realizada sempre pelas mesmas profissionais, previamente treinadas para a aplicação dos protocolos do estudo. Nenhum dos instrumentos necessitava de certificado para aplicação.

No primeiro atendimento, foi realizada entrevista com as mães/responsáveis, conduzida pela pesquisadora por meio da Ficha de Registro de Dados padronizada para este estudo, composta por questões sociodemográficas, relacionadas à gestação, nascimento, dados da internação e alta hospitalar.

A avaliação antropométrica foi realizada pela nutricionista da equipe por meio de mensuração do peso corporal, comprimento e perímetro cefálico. Para aferição do peso foi utilizada balança pediátrica digital da marca Balmak®, com capacidade máxima de 25 kg. Para mensurar o comprimento foi utilizado infantômetro de madeira com intervalo de 10 a 99 cm com subdivisões de milímetros.

A prematuridade, tratada como variável independente, foi definida por idade gestacional < 37 semanas. Também, foi classificada por meio das subcategorias: Pré-termo Extremo (< 28 semanas), Muito Pré-termo (28 a < 32 semanas), Pré-termo Moderado (32 a < 37 semanas) e Pré-termo Tardio (34 a < 37 semanas) por meio das informações registradas na Caderneta de Saúde da Criança.

Como variáveis dependentes foram consideradas:

1. Disfunção motora oral: definida como alteração funcional das habilidades orais, causadas por padrão imaturo de sucção, incoordenação entre sucção/deglutição/respiração, dificuldade na mastigação e deglutição, bem como inabilidades orais na utilização de diferentes utensílios^(2,7)^; Esta variável foi obtida por meio do Protocolo para Avaliação Clínica da Disfagia Pediátrica - PAD-PED adaptado^(8)^. Tanto nos RNPT como nos RNT, foi realizado o Exame Estrutural e Funcional do sistema sensório motor orofacial. Os RN estavam posicionados sobre uma maca em posição supino com cabeça elevada, para verificar os reflexos orais e da sucção não nutritiva. Foi utilizado o dedo mínimo enluvado na região perioral, para estimular reflexo de busca, e, em seguida, foi realizado toque na porção anterior do palato duro e na ponta da língua para eliciar a sucção.

A mobilidade e tônus de lábio, língua, bochecha foram avaliados sob observação da postura durante o repouso e da mobilidade durante desempenho das funções estomatognáticas. Este procedimento foi realizado em todas fases do acompanhamento, contemplando todas as fases do desenvolvimento.

1. Dificuldade na introdução da consistência alimentar: definida como alteração motora oral frente a diferentes consistências alimentares, diferentes sabores e utensílios^(9)^. Para esta avaliação foi utilizado o mesmo Protocolo PAD-PED adaptado^(8)^ a partir dos dados obtidos durante a oferta alimentar:

Para oferta alimentar foram utilizadas as consistências (líquido fino (leite materno/fórmula infantil), líquido espessado (leite engrossado), pastoso homogêneo (frutas/legumes amassados), pastoso heterogêneo (frutas/legumes pequenos pedaços) e sólido (frutas/legumes em pedaços) e utensílios dependendo da faixa etária. Foram ofertados pela mãe/cuidador (a) em posição usual de alimentação para oferta, respeitando o que já era introduzido pela família.

Foram consideradas dificuldades na introdução das consistências quando nos períodos em que foram realizadas a oferta das consistências, o RN ainda não as aceitava no momento da avaliação e/ou apresentava dificuldade motora oral com o mesmo, realizada por meio da observação comportamental frente à alimentação ofertada.

1. Avaliação do desenvolvimento neuropsicomotor, realizada por meio do Teste de Triagem do Desenvolvimento Neuropsicomotor Denver II (TTDD-R)^(10)^ e pela Avaliação dos Indicadores de Risco para o Desenvolvimento Infantil (IRDI)^(11)^.

Outras variáveis interferentes consideradas incluíram Avaliação da observação da amamentação, por meio do protocolo difundido pela UNICEF^(12)^, no qual é possível observar comportamentos favoráveis ao aleitamento materno, ou sugestivos de dificuldades, considerando posição corporal da mãe e do RN durante a mamada, início da mamada, eficiência da sucção, envolvimento afetivo entre o binômio, características anatômicas da mama e duração e encerramento da mamada. A partir da frequência de comportamentos desfavoráveis para cada aspecto da mamada investigada foi utilizada a classificação em Bom, Regular, Ruim^(13)^.

O estado emocional da mãe foi avaliado como outra possível variável interferente por meio da Escala de Depressão Pós-parto de Edimburgo (EDPE), a qual já foi traduzida em vários idiomas com validação em vários países, dentre eles, o Brasil. Trata-se de um protocolo de autopreenchimento que tem por finalidade a identificar e avaliar a intensidade dos sintomas de depressão pós-parto, sendo composta por 10 itens que recebem pontuação de zero a três de acordo com a intensidade relatado dos sintomas depressivos^(14)^, e a pontuação total varia de 0 a 30, sendo considerada pontuação igual ou superior a 12, sinal de depressão^(15)^.

E, por fim, foram considerados, também, os dados de baixo peso ao nascer, com peso < 2500 gramas; e os procedimentos orais invasivos como: presença e tempo de uso de nutrição enteral; presença e tempo de uso de Ventilação Mecânica; presença e tempo de uso de CPAP - *Continuos Positive Airway Pressure*, presença e tempo de uso de Tenda ou Capacete para fornecimento de O2 suplementar.

Os dados foram coletados e tabulados, exclusivamente pela pesquisadora, via planilha eletrônica *Microsoft Office Excel*® (2013), e encaminhados a um profissional habilitado para a análise estatística.

As variáveis contínuas foram avaliadas quanto a sua distribuição e estão apresentadas como média aritmética e desvio padrão, para as variáveis contínuas de distribuição normal e mediana com intervalo interquartílico (25-75%), para as de distribuição assimétrica. As variáveis categóricas estão apresentadas com suas frequências absoluta e relativa.

Para a estimativa da diferença entre variáveis contínuas foram aplicados os testes t de *Student*, teste de Mann-Whitney e Anova para medidas repetidas com teste post-hoc de Duncan.

Para estimativa da diferença entre as variáveis categóricas foi aplicado o teste exato de Fisher e o teste qui-quadrado de Pearson.

A estimativa de diferença entre as variáveis contínuas de distribuição simétrica foi realizada pelos testes t de Student e Anova para medidas repetidas com teste post-hoc de Duncan. Para as variáveis assimétricas, foi realizado o teste de Mann-Whitney. As variáveis categóricas foram avaliadas pelos testes de Fisher e qui-quadrado de Pearson.

O modelo de Regressão Logística Multivariada foi aplicado para identificar os principais fatores associados à disfunção motora oral. Considerando magnitude de efeito de 25% para o desfecho principal, qual seja proporção de RNT e RNPT com disfunção oral motora, erro do tipo I de 5% e erro do tipo II de 10%. A amostra estimada foi de 44 casos em cada grupo, conferindo poder de teste de 90% (*Statistica* v.10.0 - *Statsoft*®).

## RESULTADOS

### Características dos recém-nascidos e das mães

Durante o período do estudo foram elegíveis 153 RN que atenderam aos critérios de inclusão. Ocorreram perdas durante o estudo devido à desistência (n = 63) e devido à identificação de diagnóstico de alterações neurológicas durante o acompanhamento (n = 3). Constituíram a amostra do estudo 87 RN, dos quais, 46 constituíram o grupo de RNPT e 41, o grupo de RNT (Figura 1). Na Tabela 1 estão apresentadas as características de nascimento dos dois grupos de RN.

**Figura 1:** População do estudo

Fonte: O autor (2021)

**Tabela 1:** Características de nascimento dos recém-nascidos - Secretaria Municipal de Saúde (MAFRA-SC)

CARACTERÍSTICAS	RNT (n = 41)	RNPT (n = 46)	P
Sexo			
Masculino	18 (43,9%)	22 (47,8%)	0,82^2^
Feminino	23 (56,1%)	24 (52,2%)	
Gemelaridade	0 (0,0%)	12 (26,1%)	< 0,001^2^
Idade gestacional (semanas)	38,7 ± 1,0	34,1 ± 2,3	< 0,001^3^
Peso de nascimento (g)	3.230,0 ± 537,8	2.216,0 ± 570,2	< 0,001^3^
Classificação do tamanho ao nascimento			
PIG	10 (24,4%)	16 (34,8%)	0,12^1^
AIG	28 (68,3%)	30 (65,2%)	
GIG	3 (7,3%)	0 (0,0%)	
Perímetro cefálico (cm)	34,0 ± 1,5	31,2 ± 2,5	< 0,001^3^
Comprimento (cm)	47,7 ± 2,7	43,3 ± 3,1	< 0,001^3^
Classificação Apgar 1º minuto			
< 3	0 (0,0%)	*1 (2,2%)	0,42^1^
3-7	10 (24,4%)	15 (32,6%)	
> 7	31 (75,6%)	30 (65,2%)	
Classificação Apgar 5º minuto			
< 3	0 (0,0%)	0 (0,0%)	1,00^1^
3-7	3 (7,3%)	4 (8,7%)	
> 7	38 (92,7%)	42 (91,3%)	

1 Teste qui-quadrado de Pearson;

2 Teste exato de Fisher;

3 Teste t de Student;

* n = 1 --- = não se aplica

Legenda: PIG = Pequeno para Idade Gestacional; AIG = Adequado para Idade Gestacional; GIG = Grande para Idade Gestacional; RNT = Recém-nascidos a termo; RNPT = Recém-nascidos prematuros. Fonte: O autor (2021)

No grupo de RNPT houve maior frequência de gemelaridade (26,1% *vs* 0,0%, p < 0,001). As demais características - idade gestacional, peso ao nascimento, perímetro cefálico e comprimento foram, evidentemente, menores entre os RNPT (p < 0,001).

Nove RNT (21,9%) e 26 RNPT (56,5%) necessitaram de internação em UTIN e o tempo de permanência destes teve mediana de 6,0 (3-10) dias e de 13,5 (5-21), respectivamente. A principal causa de internação entre os RNPT foi a síndrome de desconforto respiratório 18 (75,0%).

Entre os 35 RN que necessitaram de internação em UTIN, todos os RN de ambos os grupos necessitaram de via alternativa de alimentação, entretanto o tempo de nutrição enteral foi em média significativamente maior entre os RNPT (p = 0,03). Outros procedimentos orais invasivos foram utilizados em seis RNT e em 19 RNPT. Como método de transição para via oral nos RNT o seio foi o mais utilizado e nos RNPT foi o uso de copo (p < 0,001), isto porque trata-se de uma maternidade que possui o título de Iniciativa Hospital Amigo da Criança, que utiliza este método como forma de realizar a transição e complementar a dieta dos RNPT.

No que se refere à oxigenioterapia, dos nove RNT, três necessitaram de ventilação mecânica, e, dentre os RNPT, sete (26,9%), o tempo de permanência teve uma mediana de 6 (4-10) dias e 10 (2-35) dias, respectivamente. Observou-se, também, maior frequência do uso de CPAP entre os RNPT (0% vs (57,7%), com diferença significativa, e o tempo de permanência foi uma mediana de 3 (1-6) dias.

Não foi observada diferença significativa no tipo de alimentação na alta hospitalar entre os dois grupos de RN, sendo que 100% de ambos os grupos saíram em aleitamento materno, três RNT e nove RNPT em aleitamento misto.

Observou-se, entre os RNPT, maior frequência de doença materna prévia (7,3% vs 34,8%, p < 0,001), sendo que a doença mais frequente nas mães de RNT foi a Hipertensão arterial e nas mães de RNPT, a Depressão. No que diz respeito ao histórico de saúde mental materna, 8 (19,5%) das mães dos RNT apresentaram histórico de transtorno mental e 13 (28,3%) dos RNPT, apresentaram como principal transtorno mental: Depressão.

Com relação às características dos RN relacionados aos dados antropométricos como o comprimento, perímetro cefálico e peso em todas as avaliações, estão dispostos na Tabela 2.

**Tabela 2:** Idade cronológica, idade corrigida, peso, comprimento e perímetro cefálico - Secretaria Municipal de Saúde (MAFRA -SC)

AVALIAÇÕES	RNT (n = 41)				
	Idade cronológica (dias)	Idade corrigida (dias)	Peso (gramas)	Comprimento (cm)	Perímetro cefálico (cm)
1ª avaliação	27,0 (17,50)	na	4.003,5 ± 834,8	53,4 ± 3,0	36,6 ± 2,0
2ª avaliação	4,3 ± 0,3	na	6.508,2 ± 740,4	62,0 ± 2,9	41,1 ± 1,5
3ª avaliação	6,3 ± 0,4	na	7.495,7 ± 881,2	65,4 ± 2,9	42,9 ± 1,2
4ª avaliação	9,4 ± 0,6	na	8.916,2 ± 924,0	70,4 ± 2,7	45,0 ± 1,4
5ª avaliação	12,6 ± 0,4	na	9.9917,6 ± 930,4	74,5 ± 2,2	46,3 ± 1,3
	RNPT (n = 46)				
	Idade cronológica (semanas)	Idade corrigida (semanas)	Peso (gramas)	Comprimento (cm)	Perímetro cefálico (cm)
1ª avaliação	64,0 (53-83)	21,5 (16-29)	3.949,5 ± 877,7	52,0 ± 3,8	37,2 ± 2,5
2ª avaliação	5,4 ± 0,9	4,0 ± 0,6	6.222,5 ± 962,2	60,3 ± 4,0	41,0 ± 1,9
3ª avaliação	7,6 ± 0,9	6,2 ± 0,6	7.274,8 ± 1.022,1	64,4 ± 5,1	43,7 ± 3,1
4ª avaliação	10,6 ± 1,1	9,1 ± 0,8	8.347,8 ± 1.114,1	68,9 ± 4,8	44,5 ± 2,1
5ª avaliação	13,7 ± 1,0	12,2 ± 0,8	9.258,2 ± 1.185,8	73,4 ± 5,3	46,8 ± 4,0

Legenda: RNT = Recém-nascidos a termo; RNPT = Recém-nascidos prematuros; na = não aferido. Fonte: O autor (2022)

### Protocolo de observação de amamentação

Em relação ao tipo de alimento oferecido ao RN, na alta hospitalar houve predomínio de leite materno (LM) nos dois grupos (p = 0,22). A frequência de alimentação com LM, foi maior entre os RNT na 1ª avaliação (82,9% *vs* 54,3%, p < 0,01). A partir da terceira avaliação houve queda na frequência do oferecimento de LM em ambos, sendo observado 32,4% vs 30% na última avaliação.

A frequência do uso de leite de fórmula foi semelhante entre os grupos (p > 0,05), enquanto a alimentação mista (LM + LF) foi maior entre os RNPT na 1ª avaliação (14,6% *vs* 41,3%, p = 0,01).

Durante a avaliação da amamentação observou-se, na 1ª avaliação, diferença significativa somente em relação ao item sucção entre RNT e RNPT (p < 0,01) com maior frequência de dificuldade nos RNT (27,5% *vs* 4,5%). Nas demais avaliações, em todos os itens avaliados, todos os RN dos dois grupos apresentaram classificação boa, conforme o protocolo aplicado (p = 1,00). Dificuldade na amamentação foi observada em 17 RNT (41,5%) e em 15 RNPT (34,1%) (p = 0,50) sem associação com o tipo de alimentação na 1ª avaliação (p = 0,87).

### Avaliação da alimentação

#### Histórico alimentar

Nos dois grupos observou-se modificação da posição deitada para sentada entre a 2ª e 3ª avaliação (p < 0,001). A duração da mamada foi significativamente maior entre os RNT na primeira avaliação (p < 0,01), sem diferença nas demais avaliações (p > 0,05).

Na Figura 2 está ilustrada a introdução de consistências observadas nos dois grupos de RN, isto é, quais os RN estavam aceitando no período do estudo. Na 3ª avaliação, observou-se maior frequência de administração de consistência líquida engrossada (neste caso, RN expostos a líquidos, por ex., leite engrossado) e pastosa homogênea para RNPT (84,8% *vs* 65,8%, p = 0,04). Na 4ª avaliação, foi mais frequente a administração de consistência pastosa heterogênea, também, entre os RNPT (97,0% *vs* 68,3%), p = 0,04).

**Figura 2:** Introdução das consistências alimentares - Secretaria Municipal de Saúde de MAFRA - SC

O uso de mamadeira como utensílio de alimentação foi mais frequentemente utilizado entre os RNPT na 1ª avaliação (17,1% *vs* 45,6%, p < 0,001) e 2ª avaliação (51,2% vs 73,9% p < 0,01) e o uso da colher e do copo entre os RNT na 3ª avaliação (90,2% vs 73,9% p = 0,02) e 4ª avaliação (100% vs 84,8% p = 0,02).

Na Tabela 3 estão expostas as idades em que cada consistência foi ofertada pela primeira vez aos RN (para os RNPT foi considerada a IC), bem como a duração do AME e desmame, não sendo observada diferença significativa.

**Tabela 3:** Idade dos RN em relação: ao aleitamento materno exclusivo; desmame; início da alimentação complementar e idade de introdução das diferentes consistências alimentares - Secretaria Municipal de Saúde (MAFRA -SC)

IDADE (DIAS)	RNT (n = 41)	RNPT (n = 46)	P
Do aleitamento materno exclusivo	122,0 (62-183)	122,0 (30-152)	0,04^1^
Do desmame	250,0 (150-365)	239,5 (143,5-365)	0,65^1^
Do início alimentação complementar	169,9 ± 22,7	163,1 ± 27,7	0,24^2^
Da introdução da consistência pastosa homogênea	169,9 ± 22,7	163,1 ± 27,7	0,24^2^
Da introdução da consistência pastosa heterogênea	259,6 ± 53,7	247,6 ± 51,7	0,49^2^
Da introdução de alimentos sólidos	323,1 ± 39,5	321,8 ± 51,6	0,90^2^

1 Teste de Mann-Whitney;

2 Teste t de Student.

Legenda: RNT = Recém-nascidos a termo; RNPT = Recém-nascidos prematuros. Fonte: O autor (2021)

No caso do AM exclusivo, embora as medianas sejam iguais, a variação em torno da mediana é diferente entre os grupos. Na análise estatística, considerando a dispersão, o p é de 0,04, indicando que há diferença, mas não muita, e, por isso o valor de p se aproxima de 0,05.

#### Exame estrutural e funcional

No exame estrutural e funcional dos lábios e língua, não se observou diferença entre os grupos (p > 0,05). A postura de lábios entreabertos foi observada em 37 (90,2%) dos RNT e em 37 (80,4%) dos RNPT e a postura de língua em papila em 34 (82,9%) vs 37 (80,4%), respectivamente. O tônus de língua diminuído foi observado um (2,4%) *vs* seis (13,0%).

Na avaliação da sucção não nutritiva, nos dois grupos observou-se reflexo de procura presente em 29 (70,7%) dos RNT e 25 (54,3%) dos RNPT. Não foi observada diferença significativa no padrão de sucção entre os grupos RNT e RNPT, sendo que 32 (78,1%) e 41 (89,1%) estavam adequados. A pressão intraoral estava adequada em 32 (78,1%) dos RNT e 41 (89,1%) dos RNPT.

#### Avaliação funcional com alimento

Na avaliação com alimento, em relação ao seio materno, observou-se melhor vedamento labial entre os RNT (67,5% *vs* 32,5%, p = 0,03) e melhor pega entre os RNPT (93,2% *vs* 67,5%, p < 0,001). Na relação frequência de sucção/deglutição e coordenação, não houve diferença significativa.

Na consistência líquido fino, não se observou diferença entre os grupos nos itens: vedamento labial, escape oral, coordenação e tempo de alimentação (p > 0,05).

Considerando as variáveis: coordenação, pega, vedamento labial, escape pelas comissuras labiais e sucção inadequada, a DMO foi observada em 15 casos (36,6% *vs* 32,6%) em cada grupo na primeira avaliação. Dentre os RN com DMO, houve persistência em dois casos entre os RNT e três casos entre os RNPT na última avaliação, sendo observado nestes últimos, dificuldade no preparo e mastigação para as consistências pastoso heterogêneo e sólido, escape anterior de líquido em copo aberto. Na Tabela 4 estão apresentadas as principais características dos 5 RN que apresentaram DMO na última avaliação.

**Tabela 4:** Principais características dos recém-nascidos com disfunção motora oral na última avaliação - Secretaria Municipal de Saúde (MAFRA-SC)

N	GRUPO	APGAR	CP	POI	IT	MAMAD	DF AM	SUCÇÃO DEFICIENTE	DMO 7-15 DIAS	DENVER	RISCO PSÍQUICO	IRDI
1	RNPT	9/10	Sim	Sim	Não	Não	Sim	Sim	Sim	Normal	Não	Alterado
2	RNPT	7/9	Sim	Sim	Sim	Sim	Não	Não	Sim	Normal	Não	Normal
3	RNPT	4/7	Sim	Sim	Não	Não	Sim	Não	Sim	Normal	Não	Normal
4	RNT	9/10	Sim	Sim	Sim	Não	Sim	Não	Sim	Anormal	Não	Normal
5	RNT	8/8	Não	Não	Não	Não	Não	Não	Não	Normal	Não	Normal

Legenda: RNT = Recém-nascidos a termo; RNPT = Recém-nascidos prematuros; APGAR = Índice de APGAR, é um teste feito no recém-nascido logo após o nascimento que avalia o seu estado geral e vitalidade; CP = Diagnóstico cardiopulmonar; POI = Procedimento oral invasivo; IT = Intubação traqueal; MAMAD = Mamadeira na alta hospitalar; DF AM = Dificuldade na amamentação; DMO = Disfunção motora oral; IRDI = Índice de risco de desenvolvimento infantil Alterado: 2 ausências ou mais. Fonte: O autor (2021)

Dentre as principais diferenças entre os RN com DMO a termo e prematuros estão a gemelaridade (0% *vs* 33,3%), internação em UTIN (33,3% *vs* 80%), uso de CPAP (0% *vs* 46,7%) e de SNG (13,3% *vs* 73,3%). Além disso, os RNT apresentaram mais frequentemente dificuldade no aleitamento materno e sucção ruim (p < 0,01) com diferença significativa neste último.

Considerando a análise de todos sem DMO (n = 57) e com DMO (n = 30), observou-se que não houve diferença em relação à frequência de prematuridade (p = 0,82) e anormalidades do desenvolvimento neuropsicomotor (DNPM) (p = 0,20) entre os grupos. As variáveis associadas a DMO foram: a dificuldade no aleitamento materno, a resposta à amamentação, sucção deficiente, internação em UTIN, procedimentos orais invasivos e uso de mamadeira na alta hospitalar (Tabela 5).

**Tabela 5:** Principais diferenças dos recém-nascidos com disfunção motora oral na 1ª avaliação - Secretaria Municipal de Saúde (MAFRA-SC)

CARACTERÍSTICAS	SEM DMO (n = 57)	COM DMO (n = 30)	p
Idade gestacional	36,5 ± 2,4	35,9 ± 3,7	0,34^1^
Prematuridade	31 (54,4%)	15 (50,0%)	0,82^2^
Peso ao nascimento	2728,5 ± 672,0	2628,0 ± 890,5	0,55^1^
Internação UTI	18 (31,6%)	17 (56,7%)	0,03^2^
Intubação traqueal	2 (3,6%)	8 (26,7%)	0,02^2^
Procedimentos orais invasivos	18 (31,6%)	17 (56,7%)	0,03^2^
Dificuldade amamentação	15 (26,3%)	17 (56,7%)	< 0,01^2^
Resposta	2 (3,6%)	9 (30,0%)	< 0,001^2^
Sucção	3 (5,4%)	14 (46,7%)	< 0,001^2^
Mamadeira na alta	3 (5,3%)	9 (30,0%)	< 0,01^2^
Anormalidade Denver	8 (14,0%)	7 (23,3%)	0,20^2^

1 Teste t de Student;

2 Teste exato de Fisher

Legenda: DMO = Disfunção Motora Oral; UTI = Unidade de Terapia Intensiva. Fonte: O autor (2021)

Na análise dos principais fatores preditivos para DMO, por meio de regressão logística multivariada, o uso de mamadeira elevou a chance de DMO em cerca de 7 vezes (OR = 7,55, IC 95% = 1,66-34,18, p < 0,01) e a ocorrência de procedimentos orais invasivos em cerca de 5 vezes (OR = 4,95, IC 95% = 1,09-22,27, p = 0,02) não sendo observada mesma relação com os indicadores: internação em UTIN, dificuldade na amamentação e sucção.

### Avaliação do neurodesenvolvimento

Na avaliação do desenvolvimento pelo teste de Denver observou-se maior frequência de casos classificados como anormal entre os RNPT na 2ª e 3ª avaliações.

Dentre os itens avaliados no protocolo no componente pessoal-social, observou-se na 1ª avaliação maior frequência de classificação suspeita e anormal entre os RNPT (suspeito: 15,2% *vs* 2,4%; anormal: 8,7% *vs* 2,4%, p = 0,04). Não se observou diferença significativa na classificação da linguagem entre os grupos em nenhuma das avaliações (p > 0,05).

No componente motor fino não se observou diferença entre os grupos (p > 0,05). Para o componente motor grosso na 2ª avaliação (suspeito: 30,4% *vs* 12,2%; anormal: 21,7% *vs* 7,3%, p < 0,001) e 3ª (suspeito: 10,9% *vs* 2,4%; anormal: 28,3% *vs* 2,2%, p = 0,03) observou-se maior frequência de casos suspeitos e anormais entre os RNPT.

Ao realizar associação entre os registros do protocolo Denver II e a avaliação de DMO observou-se que na 1ª avaliação, em três casos registrou-se atraso no Denver no item Motor Grosso, um deles com DMO; na 2ª avaliação, 18 casos, nenhum com DMO; na 3ª, 13 casos, nenhum com DMO e na 4ª, sete casos, um com DMO, sem diferença entre RNT e RNPT, não se evidenciou associação entre disfunção motora grossa e DMO (p > 0,05).

### Avaliação dos Indicadores de Risco para o Desenvolvimento Infantil (IRDI)

Não se observou diferença significativa entre os indicadores de risco para o desenvolvimento infantil em todas as avaliações (p > 0,05). Contudo identificou-se o risco psíquico em 2,2% somente para o grupo de RNPT (p = 1,00).

### Triagem do estado emocional da mãe

Para a triagem do estado emocional da mãe foi utilizada a escala de Depressão Pós-parto de Edimburgo, com presença de sinais de depressão em 11 casos no grupo de RNT (26,8%) e sete casos no grupo de RNPT (15,2%) (p = 0,19) na 1ª avaliação. Na 2ª avaliação, estas frequências se reduziram a 7,3% (três casos) e 2,2% (um caso) (p = 0,33), tendo sido todas encaminhadas para orientação psicológica e não houve casos nem persistência de sintomas nas avaliações seguintes.

## DISCUSSÃO

O principal resultado deste estudo, que buscou avaliar a presença de DMO em RNPT e comparar com RNT, bem como avaliar se há correlação entre DMO e dificuldade na introdução da consistência alimentar nestas populações, foi que, embora a DMO tenha sido observada, não houve diferença entre RNPT e RNT. Além disso, apesar da ocorrência de DMO, não houve dificuldade no período de início da alimentação complementar, que ocorreu de modo precoce. A DMO foi observada em 15 casos, em cada grupo (RNT e RNPT, respectivamente) na primeira avaliação e houve persistência da DMO em dois casos dos RNT e três casos entre os RNPT na última avaliação. Foi observado nestes últimos, dificuldade no preparo e mastigação para as consistências pastoso heterogêneo e sólido, correspondendo a dificuldade para estas consistências e escape anterior de líquido em copo aberto, correspondendo a dificuldade oral com este utensílio.

A real prevalência dos problemas de deglutição e de DMO em neonatos e lactentes não é conhecida. Estudos demonstraram que na avaliação de RNPT aos 4 meses, a presença de DMO variou entre 23% a 89% para a consistência pastosa; aos seis meses, identificaram DMO em aproximadamente 40% dos RNPT da amostra para a consistência semissólida e aos 12 meses uma variação entre 8% a 28% para a consistência sólida^(6,16-18)^.

Nesses estudos, a IG ao nascimento foi em média de 32 semanas, enquanto que no presente estudo foi de 34 semanas. Vale ressaltar que esta diferença de duas semanas de IG traduz-se numa diferente evolução neurofisiológica, isto é, há grande diferença na maturidade neurológica e, consequentemente, são observados diferentes níveis nas habilidades motoras orais

No presente estudo, a DMO não apresentou relação com atrasos no desenvolvimento neuropsicomotor avaliados pelo Denver II, porém os pacientes de alto risco neurológico foram excluídos da amostra. Além disso, os pacientes foram acompanhados por uma equipe interdisciplinar, e as orientações necessárias quanto à alimentação e nutrição, além de intervenções psicossociais, foram realizadas a cada avaliação. Portanto, um risco de viés de intervenção pode ter sido responsável pela baixa frequência de alterações no presente estudo. Além disso, a população deste estudo era predominantemente de RNPT tardios e não de prematuros extremos, esta mais predisposta ao risco de DMO que aquela.

A prevalência de problemas de alimentação descrita em um estudo populacional no Reino Unido em 2001, com 14.000 RNPT nascidos com <37 semanas de gestação foi de 10,5% e essa frequência aumentou para 24,5% entre aqueles nascidos com muito baixo peso (<1500 g)^(19)^. Esse estudo, também, relaciona alterações alimentares persistentes nos primeiros 15 meses de vida com atraso no desenvolvimento neuropsicomotor. Já as dificuldades alimentares nas primeiras quatro semanas de vida são muito frequentes e não têm um valor preditivo importante.

Na análise dos 30 RN (15 em ambos os grupos) com DMO, não foi observado relação com a prematuridade e desenvolvimento neuropsicomotor, mas foi possível verificar a associação com dificuldade de aleitamento materno, internação em UTIN, procedimentos orais invasivos neonatais e uso de mamadeira na alta hospitalar.

Estudos apontam que os RNPT são expostos a estímulos externos prolongados e nocivos, como cânulas orotraqueais e sondas orogástricas e que tais intervenções podem impactar negativamente as habilidades orais desta população^(1,20,21)^, com potencial risco de aversão à alimentação oral a médio e longo prazo^(1,17)^.

Com relação aos cinco RN com DMO na última avaliação, quatro passaram por intervenções orais invasivas, e já apresentavam DMO na primeira avaliação. Quando realizada a análise dos preditores para DMO, a ocorrência de procedimentos orais invasivos aumentou em 6 vezes a chance de apresentar tal dificuldade.

Em um estudo populacional no Reino Unido, por meio de questionário aplicado por telefone, comparando 1130 RNPT com 1255 RNT, os RNPT apresentaram mais dificuldades alimentares aos dois anos. O risco relativo de dificuldades alimentares foi 1,57 e 1,62 para DMO e o uso de sonda nasogástrica por mais de 2 semanas foi associado a dificuldades alimentares^(22)^.

Em outro estudo transversal brasileiro de 62 RNPT, também, associou tempo de sonda enteral com dificuldades alimentares e comportamentos defensivos aos 13 meses de idade corrigida. Mas não encontrou associação entre DMO e dificuldade alimentar^(6)^.

Outros dois estudos relatam associação significativa entre dificuldades alimentares e IG, em tais estudos a população estudada foram RNPT extremos^(5,6)^. Já outro estudo transversal, também, com RNPT com média de IG de 32 semanas, não encontraram relação entre DMO e IG^(16)^, como no presente estudo. Como já observado, a população deste estudo foi de RNPT tardios, o que pode explicar a não associação com IG.

O período de introdução da alimentação complementar, bem como a idade apropriada de início da alimentação oral com a exposição a texturas e sabores respeitando as janelas de oportunidade e todos os estímulos e experiências que envolvem a relação com o alimento e com o desenvolvimento das competências oro motoras, podem ter envolvimento nas dificuldades de alimentação em RNPT a médio e longo prazo^(2)^.

Os resultados da presente pesquisa demonstraram que tanto nos RNT quanto nos RNPT, a introdução da consistência pastosa ocorreu precocemente, por volta dos quatro meses, corroborando com estudos realizados com prematuros, no qual os mesmos foram expostos à oferta de frutas/papas antes de completar seis meses de idade corrigida^(5,6,18,20,22-25)^.

A consistência pastosa heterogênea (pequenos pedaços) foi observada com maior frequência na 4ª avaliação, na qual os RN tinham idade média de oito meses (idade corrigida RNPT). Já a consistência sólida foi iniciada em ambos os grupos, por volta dos dez meses, conforme esperado para a idade. Entretanto foi observada uma pequena parcela de RN que na 5ª avaliação, ainda não aceitavam nem pequenos pedaços nem sólidos, sendo considerado um atraso na introdução dos mesmos.

Quando o assunto é a introdução das consistências, a literatura traz como balizadoras as idades de janelas de oportunidade. No sexto mês de idade corrigida é iniciada a alimentação complementar que deve ocorrer de forma gradual na consistência pastosa, aos oito meses, o lactente já está apto a receber alimentos em pequenos pedaços e/ou desfiados. Esta não deve passar dos nove meses, podendo ocasionar futuramente problemas alimentares e a introdução para consistência sólida igual da família, deverá ser realizada até os 12 meses^(6,9)^.

Além disso, King^(9)^ reforça que a introdução da alimentação complementar deve respeitar as competências e o ritmo dos RNPT, para que desenvolvam as habilidades adequadas a cada progressão de textura.

A oferta de alimentos, água, chás e sucos antes dos seis meses de vida, já caracteriza a introdução precoce de alimentação complementar^(25)^. Tanto o Ministério da Saúde quanto a Sociedade Brasileira de Pediatria não recomendam esta prática, pois pode acarretar em diminuição do aleitamento materno exclusivo ou até seu desmame^(26,27)^.

Apesar da recomendação do Ministério da Saúde (2010) de que o leite materno deve ser exclusivo até os seis meses de vida para a população de RNPT. A literatura ainda é escassa e não há consenso, entretanto há a recomendação para que a introdução da alimentação complementar se inicie a partir de seis meses de idade corrigida, sendo que, também, devem estar presentes os sinais de prontidão^(28)^.

Vale ressaltar que quando a introdução da alimentação complementar é iniciada precocemente, a criança pode desenvolver doenças alérgicas ou até mesmo alteração no desenvolvimento oral, implicando em dificuldades na mastigação. Já quando ela ocorre tardiamente, poderá ocorrer um déficit de crescimento ou anemia, e comprometer o crescimento e desenvolvimento das estruturas faciais^(5)^.

No que diz respeito à média de internação em UTIN, o tempo de permanência depende da complexidade e do grau de prematuridade dos pacientes atendidos no serviço. A maioria dos estudos com UTIN de alta complexidade descrevem tempo médio de internação dos RNPT e BPN acima de um mês^(29)^. Em um estudo, os RNs necessitaram de internação em UTIN 18 (31,6%) RNT *vs* 17 (56,7%) RNPT. A média do tempo de internação em UTIN foi de 6 (3-10) dias no grupo dos RNT e de 13,5 (5-21) dias no grupo de RNPT, esta mais suscetível a intercorrências peri e pós- natal, necessitando, assim de cuidados intensivos^(6)^. Este dado demonstra que os RNPT da maternidade envolvida no serviço não eram pacientes de alta complexidade. Demonstra, também, que muitos RNT do grupo estudado, ao invés de receberem alta com 48h a 72h, permaneceram internados por mais tempo, o que indica que a população de RNT estudada, apesar de não ter o risco da prematuridade, tinha outros fatores de risco, por exemplo, internação em UTIN.

Um viés de amostragem importante de se relatar foi que os RNT também eram considerados de risco, além de alguns necessitarem de internação em UTIN. Outros foram selecionados a partir de dificuldades no aleitamento materno durante a realização do teste do pezinho, sendo encaminhados ao NASF para avaliação especializada. De qualquer modo, a DMO foi observada, também nos RNT, o que indica que mesmo neste grupo teoricamente com prognóstico favorável, de acordo com a idade gestacional, o risco existe, e uma avaliação e triagem especializada devem ser consideradas, principalmente, naqueles mais expostos à internação em UTIN.

No presente estudo, 9 (21,9%) RNT passaram por internamento em UTIN, permanecendo um tempo de médio de 6,0 (3-10) dias. A maioria destes teve como causa de internamento diagnóstico cardiopulmonar. Embora não tenham sido observadas alterações na Triagem do Desenvolvimento, há de se considerar que tal diagnóstico pode acarretar em atrasos no desenvolvimento motor fino.

Na alta hospitalar, a frequência de AM foi elevada: 100% nos RNT e 97% nos RNPT, mista em 7,3% e em 19,6%, respectivamente. A Iniciativa Hospital Amigo da Criança tem desempenhado papel valioso na mobilização dos atores envolvidos dentro das instituições hospitalares, no processo de mudança de condutas e rotinas aos elevados índices de desmame precoce^(21)^.

A Maternidade Dona Catarina Kuss é uma instituição que faz parte da Iniciativa Hospital Amigo da Criança (IHAC) e possui o Método Mãe Canguru e ambos propiciam, incentivam e promovem o aleitamento materno^(25)^. As mães dos RNPT permanecem internadas juntamente com seus filhos e são capacitadas e treinadas para o AM, por meio de auxílio, estratégias e intervenções que o promovem com efetividade e segurança antes da alta.

Embora exista e Estratégia Amamenta e Alimenta Brasil que são ações para o fortalecimento da promoção, proteção e apoio ao aleitamento materno e a alimentação complementar saudável para crianças menores de dois anos de idade^(21)^, os números do município estão muito aquém do esperado, entre a alta hospitalar até a 1^a^ avaliação, foi observada diminuição da frequência de aleitamento materno em ambos os grupos, entretanto esta frequência foi ainda menor nos RNPT, passando a serem semelhantes nas demais avaliações, há de se pensar em estratégias que possam estreitar a assistência, a fim de proteger o aleitamento materno.

No que diz respeito às características da avaliação da mamada foi observada diferença significativa no item sucção, com maior frequência de dificuldade nos RNT. Sendo observado melhor vedamento labial no RNT e melhor pega nos RNPT. Uma hipótese para este dado seria por que os RNT foram selecionados para este estudo a partir da solicitação de avaliação e manejo do aleitamento materno, isto é, apresentavam algum grau de dificuldade ou queixa durante o aleitamento materno, enquanto os RNPT já vinham da maternidade com estas questões melhores estabelecidas. Este dado enfatiza que as dificuldades com o aleitamento materno não ocorrem apenas na população de RNPT, pelo contrário, talvez alguns RNT precisassem de mais tempo e de maior suporte e apoio profissional para estabelecimento do AM, pois os RNT expostos a fatores de risco para o desenvolvimento, também, merecem receber avaliação e acompanhamento especializado.

É importante ressaltar que o leite materno é o melhor e mais completo alimento para o recém-nascido, seja ele prematuro ou a termo, e que após a introdução da alimentação complementar é recomendada a continuidade do AM até os dois anos de vida ou mais^(25)^. O tempo de Aleitamento Materno Exclusivo foi abaixo do preconizado 180 dias (seis meses), em ambos os grupos com idade média de 122 dias (quatro meses) e a média de idade de desmame do AM foi de oito meses nos RNT e de sete meses de idade corrigida nos RNPT.

Além de questões orgânicas, emocionais e ambientais, quando se trata de Aleitamento Materno Exclusivo (AME), há de se considerar que atualmente a Consolidação das Leis do Trabalho (CLT) não caminham paralelamente às recomendações da Organização Mundial da Saúde (OMS), com licença maternidade de quatro meses. Isso somado a pouca ou restrita rede de apoio, instituições/creches que não comportam a oferta de leite materno, vulnerabilidade social, entre outros, favorecem os baixos índices de AME.

Nos resultados dos Indicadores de Risco para o Desenvolvimento Infantil (IRDI), apesar de serem observadas algumas ausências de 2 ou mais itens, a maioria dos indicadores estavam presentes, e quando ausentes foi realizada intervenção interprofissional e reavaliados na consulta seguinte. Além disso, vale ressaltar que para ser considerado risco psíquico é necessário que os indicadores ausentes persistam na 2ª avaliação. Tais resultados podem estar relacionados às características da própria Maternidade Dona Catarina Kuss, da qual os RN eram provenientes, além do fato de estarem todos inseridos num Serviço de Seguimento ao RN de Risco para o Desenvolvimento orientado, dentre outros eixos, à promoção de saúde mental materno-infantil.

Em relação à amostra deste estudo, o seguimento prolongado proposto foi acometido por diversas variáveis que culminaram em relevante perda de seguimento. Preocupados com a perda de seguimento nos estudos de coorte prospectivos na primeira infância, pois trata-se de um problema enfrentado em todo o mundo, Keys e colaboradores^(30)^ estão conduzindo uma pesquisa de revisão sistemática no Canadá com o objetivo de levantar os elementos que levam a falhas no recrutamento e retenção de pais nos estudos e centros de seguimento de crianças de 0 a 36 meses. Objetivam ao término da pesquisa, oferecerem recomendações para que pesquisas futuras adotem estratégias mais eficientes de recrutamento e, especialmente, de retenção dos participantes nesta população. De modo geral, estudos com amostras maiores se fazem pertinentes para a avaliação das variáveis descritas neste estudo, idealmente com RNT saudáveis e sem intervenção e com uma população grande de RNPT extremos. Para a real prevalência de DMO, estudos preferencialmente sem viés de intervenção são necessários. Porém como a avaliação da DMO é feita por especialistas da fonoaudiologia, uma avaliação livre de intervenção seria contrária a uma conduta adequada.

## CONCLUSÃO

Não foi observado diferença na introdução das consistências no período de início da alimentação complementar entre RNT e RNPT. A consistência pastosa foi introduzida precocemente em ambos os grupos.

Dificuldades com aleitamento materno ocorreram apenas na primeira avaliação e em uma pequena proporção, não havendo diferença entre RNT e RNPT.

A frequência de AM foi maior entre os RNT na 1ª avaliação, sendo semelhante a partir das demais avaliações, entretanto tanto o AME quanto o desmame estão muito aquém do preconizado em ambos os grupos.

A DMO ocorreu numa pequena parcela de ambos os grupos na primeira avaliação (RNPT e RNT, respectivamente) e não havendo persistência de dificuldade nas diferentes consistências no período de início da introdução alimentar. E na última avaliação a DMO foi observada numa frequência menor que na primeira, sendo observado atraso na introdução de sólidos em alguns casos.

Com relação aos preditivos para DMO, foi observado que a mamadeira elevou a chance de DMO em cerca de 7 vezes, e os procedimentos orais invasivos em cerca de 6 vezes.

Não foi observado associação entre Desenvolvimento Neuropsicomotor com DMO e AM.

Não foi observado associação entre depressão materna e AM.
